# Potential Prognostic Value of a Seven m6A-Related LncRNAs Signature and the Correlative Immune Infiltration in Colon Adenocarcinoma

**DOI:** 10.3389/fgene.2021.774010

**Published:** 2021-12-22

**Authors:** Xiu-kun Chai, Wei Qi, Chun-Yan Zou, Chen-Xi He, Miao Su, Dong-Qiang Zhao

**Affiliations:** ^1^ Department of Gastroenterology, The Second Hospital of Hebei Medical University, Shijiazhuang, China; ^2^ Department of Gastroenterology, Qinhuagdao First Hospital, Qinhuangdao, China; ^3^ Department of Gastroenterology, Xingtai City People's Hospital, Xingtai, China; ^4^ Department of Gastroenterology, Harrison International Peace Hospital, Hengshui, China

**Keywords:** lncRNA, m6A, bioinformatics analysis, immune cell infiltration, COAD

## Abstract

Long non-coding RNAs (lncRNAs) and their N6-methyladenosine (m6A) modifications play an essential role in tumorigenesis and cancer progression. This study was designed to explore the value of m6A-related lncRNAs in prognosis and therapeutic applications of immune infiltration of colon adenocarcinoma (COAD). We downloaded the COAD gene expression and clinical data from The Cancer Genome Atlas project. By co-expression analysis, Lasso Cox regression analysis, and univariate and multivariate Cox regression, we constructed an independent prognostic signature of seven m6A-related lncRNAs. The prognostic lncRNAs were divided into two clusters by consistent clustering analysis, as well as into two groups of low–high risk based on the signature. Then we identified the relationship between the different groups with clinical features and immune cell infiltration. Cluster 2 had a higher risk score with a lower survival rate. The risk score was higher in groups with advanced clinical features, such as stage III–IV, N1-3, and M1. The expression of AC156455.1 was increased in tumor tissues and cluster 2, and the lncRNA ZEB1−AS1 was notably higher in the high-risk group. Five types of immune cells showed differences in two clusters, and most were upregulated in type 2. The expression of memory B cells was positively correlated with the risk score. The prognostic model was verified by the Gene Expression Omnibus (GEO) dataset. Besides, we found that the expression of these seven lncRNAs in tumor tissues was significantly higher than that in normal tissues, which verified the feasibility of the model. Thus, the signature of seven m6A-related lncRNAs can independently predict the prognosis of COAD. This signature is also closely associated with immune cell infiltration, and new therapeutic targets can be explored from this field.

## Introduction

Colorectal cancer (CRC) is one of the most common and malignant tumors, ranking third in incidence and second in mortality from cancer-related deaths worldwide (Siegel et al., 2021). Colon adenocarcinoma (COAD) accounts for more than 90% of different pathological subtypes of it. So far, the general treatment of surgical resection and combination therapy, such as radiotherapy, chemotherapy, and immunotherapy, remains the only effective therapy for COAD.

Treatment techniques are improving, but the results are unsatisfactory, with a 5-year relative survival rate of 90% of localized CRC to 14% of metastasized (Kweon, 2018). First, more than 50% of the patients with CRC have liver metastasis for the late diagnosis (Banerjee et al., 2017). Second, the recurrence rate remains high after surgery. Third, the benefits of radiation and chemotherapy remain low, resulting in few effective treatments for patients in an advanced stage. Therefore, new and efficient prognostic markers and therapeutic targets must be discovered urgently.

The occurrence and development of tumors are closely related to the changes of genes and epigenetic changes. As the most abundant apparent epigenetic modification, N^6^-methyladenosine plays a crucial part in nearly all stages of RNA transcription, processing, degradation, and translation. There exist three types of m^6^A regulators, including methyltransferase “writers” (such as METTL3, METTL14, etc.), demethylase “erasers” (FTO and ALKBH5), and m^6^A binding protein “readers” (YTHDC, YTHDF1/2/3, etc.) (Zaccara et al., 2019). Recently, increasing studies have demonstrated that aberrant m^6^A modifications play an important role in the occurrence and progression of tumors, including COAD, and act as the tumor suppressor (Li et al., 2019; Yue et al., 2019). Gu et al. discovered that through an m^6^A-dependent manner DMDRMR could interact with IGF2BP3 to regulate target genes and act as a prognostic and therapeutic target for clear cell renal cell carcinoma (ccRCC) (Gu et al., 2021).

Researchers found that, consisting of more than 200 nucleotides, long non-coding RNAs (lncRNAs) significantly impact the epigenetic modifications, regulation, and splicing (Li et al., 2017). They can regulate physiological processes such as cell differentiation, immune response, and apoptosis (Liu et al., 2016). With more and more lncRNAs identified, 14,826 lncRNAs have been annotated by the GENCODE consortium (v22; https://www.gencodegenes.org/). Emerging evidence suggests that lncRNAs participate in regulating proliferation and metastasis of various tumor cells and could be used as diagnostic and prognostic markers (Zhu et al., 2017; Kong et al., 2019; Poursheikhani et al., 2020). Gutschner et al. found the lncRNA MALAT1 could act as a prognostic marker of lung cancer and the knockout of MALAT1 related to little metastasis (Gutschner et al., 2013). Cen et al. discovered that lncRNA IGFL2-AS1 could result in a pretty poor prognosis for the patients of COAD (Cen et al., 2021).

In recent years, research on the relationship between m^6^A methylation and lncRNA in tumors has become a hotspot, showing that m^6^A-related lncRNA can serve as novel potential prognostic targets for multiple cancers (Jin et al., 2021; Li et al., 2021b; Liu et al., 2021). Romanowska et al. identified a risk signature including four m^6^A-related lncRNAs for head and neck squamous cell carcinoma (HNSCC) patients (Romanowska et al., 2021). Yu et al. constructed a prognostic signature based on m^6^A-related lncRNAs to predict the prognosis accurately of renal clear cell carcinoma patients (Yu et al., 2021). However, the study of COAD-related m^6^A methylation based on lncRNA remains relatively few.

Multiple studies have shown that the tumor microenvironment (TME) relates closely to the prognosis and treatment effect of tumors, for playing a vital role in the progression (Danaher et al., 2018; Toor et al., 2020), as well as in immunotherapies of COAD (Koi and Carethers., 2017). Although therapies are improving, the 5-year survival rate of advanced COAD is only 10% for chemotherapeutic drug resistance and disease recurrence.

Therefore, the finding of novel therapeutic markers and strategies for COAD is crucial. Thus, we performed the study to explore the relationship between m^6^A-related lncRNA and COAD to find new prognostic markers and therapeutic drug targets.

## Materials and Methods

### Acquisition and Preprocessing of COAD Data

**FIGURE 1:**
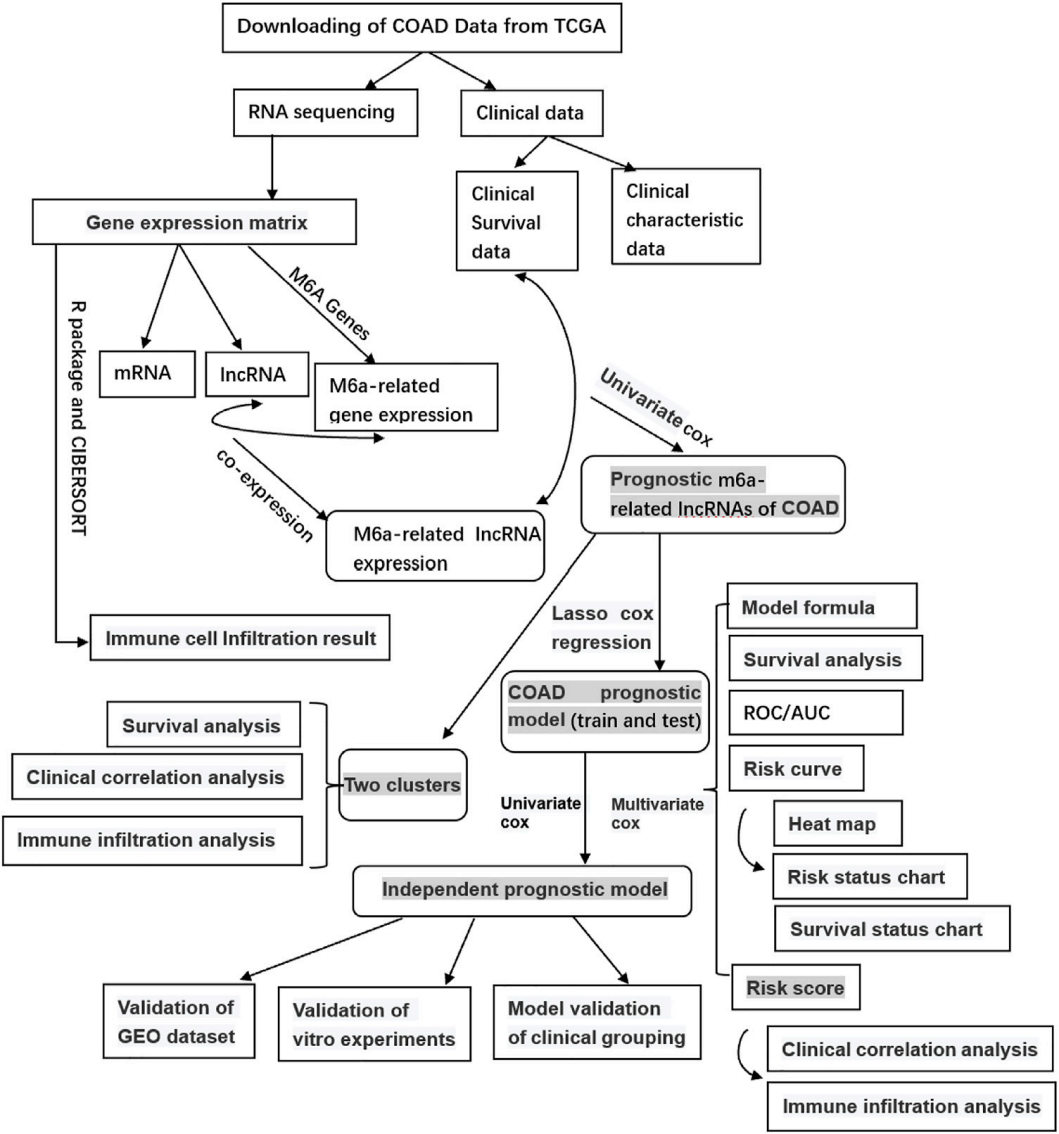
Study flow chart of the integration analysis.

We obtained the RNA sequencing (RNA-seq) of COAD from the database of The Cancer Genome Atlas (TCGA) (https://portal.gdc.cancer.gov/), including 39 healthy samples and 398 tumor samples. Meanwhile, we collected clinical data grouped according to survival time, survival status, gender, age, stage, and TMN stages. Then, using Perl software, we obtained the mRNA expression matrix and the gene expression matrix based on the human genome annotation data downloaded from the GENCODE website (https://www.gencodegenes.org/human/). Then we got the RNA and lncRNA expression as well as the m^6^A-related gene expression based on the m^6^A-related genes on the web and R language software. Finally, the m^6^A-related lncRNA expression was obtained by co-expression analysis of lncRNA and the m^6^A-related gene expression using the R package (limma, corFilter = 0.4, *p* = 0.001). The workflow is shown in Figure 1.

### Identification of Prognostic m^6^A-Related LncRNAs

The clinical survival data, containing only survival time and survival state data, were combined with m6A-related lncRNA expression data. Then using the R survival package and the univariate Cox analysis (if *p* < 0.05, there were too many lncRNAs, set *p* < 0.001), prognostic m6A-related lncRNAs would be obtained. The confidence interval and the hazard ratio were calculated using the survival package and visualized by forest plots. Differences in prognostic m6A-related lncRNAs in normal and tumor samples were analyzed by differential analysis. The results were obtained through R packages (limma, pheatmap, reshape2, ggpubr), and a boxplot and heat map were prepared to visualize the results.

### Relationship Between Clusters With Clinicopathological Features and Immune Cell Infiltration

Firstly, the prognostic m^6^A-related lncRNAs were classified into two clusters based on the packages, Limma and ConsensusClusterPlus, according to the lncRNAs expression. By the consistent cluster analysis, we calculated the cluster Max K value = 9, clusterNum = 2. Using survival and survminer packages, survival analysis of the two prognostic m^6^A-related lncRNAs types was performed, and the survival curves were drawn. Subsequently, we analyzed the correlation between the two subtypes and clinical characteristics and visualized them by drawing heat maps. The immune cell infiltration results were obtained using CIBERSOERT, and the R packages BiocManager, Limma, PreprocessCore, and E1071. The TME was scored using the estimate, limma, and BiocManager packages. Then we obtained the immune cell, stromal, and estimate score files and compared their differences in two clusters. Explore differences of the immune cells in different types by Limma and Vioplot packages.

### Construction of Independent Prognostic Model and the Risk Groupings

Prognostic m^6^A-related lncRNA samples were divided into training and testing datasets. By using caret, glmnet, survminer, and timeROC packages, also applying Lasso regression, the optimal prognostic model and model formula were obtained. The two groups were sorted into two groups of high and low risk, according to the median risk score of the training dataset. Then, survival analysis of the two groups was performed by survival and survminer packages. All *p*-values were less than 0.01, indicating differences in survival, suggesting that the model can divide patients into high- and low-risk groups. Setting the prediction time at 1 year, we drew the receiver operating characteristic (ROC) curves by survival, survminer, and timeROC packages. Then, risk graphs of risk state, survival state, and risk heat map were obtained. Through univariate and multivariate Cox regression analysis, we confirmed whether the risk score was an independent indicator of the prognosis and whether the prognostic model was independent.

### Relationship Between Risk Score With Clinicopathological Features and Immune Cell Infiltration

The model would be verified to be applied to patients in different clinical groups by survival and the survminer R package. We applied limma, ggpub, and pheatmap packages to explore differences between risk scores with clinicopathological characteristics, immune scores, different clusters, and even the seven lncRNAs, visualized by the R package (*p* < 0.001***, 0.01**, 0.05*). Then we used limma, ggplot2, ggpubr, and ggextra packages to identify the correlation between immune cells and the risk score. Samples with *p* > 0.05 would be filtered out before analysis, and the sum of all immune cells in a sample is equal to 1. The correlation coefficient and *p*-value were obtained by the Spearman test, visualized by scatter plots.

### Validation of the Prognostic Value of Seven Screened m^6^A-Related LncRNAs by Gene Expression Omnibus Dataset

To verify the prognostic values of the seven m6A-lncRNAs signature, we gained external cohort (GSE39582) from Gene Expression Omnibus (GEO) (https://www.ncbi.nlm.nih. Gov/geo/query/acc.cgi). Calculating the risk-score of every patient based on the signature, we classified the subjects into high- and low-risk groups with the median score of the training dataset of TCGA data. Through the survival package of R, we performed the survival probability. Therefore, the prediction model could be validated.

### Validation of the Expression Level of Seven m^6^A-Related LncRNAs in Tissue Specimens by Quantitative Reverse Transcription-PCR

We collected 20 paired COAD samples and tumor-adjacent normal tissues from the Second Hospital of Hebei Medical University. All the fresh samples were frozen in liquid nitrogen immediately and stored at −80°C. All tissues were confirmed histopathologically by pathologists and did not receive preoperative radiotherapy or chemotherapy. All patients signed the informed consent before the operation, and the ethics committees approved the consent procedure. The expression levels of the seven lncRNAs were examined by quantitative reverse transcription (qRT)-PCR. The 2^−ΔΔCt^ method was used to calculated the relative expression level.

### Statistics

All analyses were performed by R software v4.1 (https:// www.r-project.org/) and Perl v5.30 (https://www. perl.org). The *p*-value used in the analysis was two-sided, and a *p*-value <0.05 was regarded as statistically significant. Univariate Cox regression, multivariate Cox regression, and Lasso regression were utilized. Quantitative data were expressed as mean ± SD and analyzed for significant difference in two groups by Student's t-test. All data with *p*-values less than 0.05 were recognized as statistically significant.

## Results

### Nine Prognostic m^6^A-Related LncRNAs

**FIGURE 2:**
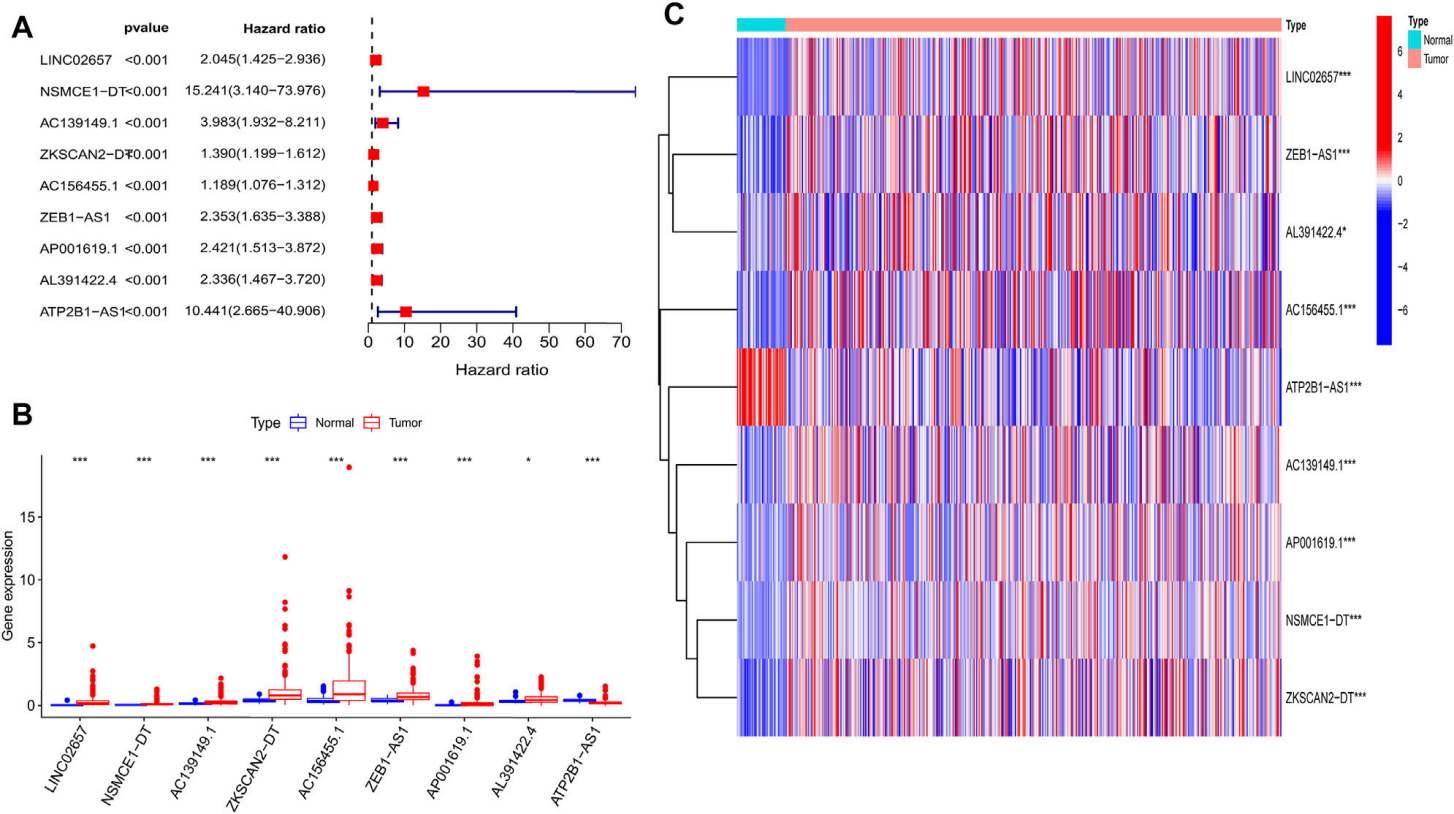
Nine prognostic m^6^A-related lncRNAs of COAD. **(A)** Forest map of nine prognostic m6A-related lncRNAs by univariate Cox regression analysis. Red represents high risk, while green represents low risk; all of them were high risk. **(B)** Boxplot of the different expressions of the nine prognostic related lncRNAs among tumor and normal tissue; the expression of AC156455.1 in tumor tissue was significantly higher than that of the normal group. **(C)** Heat map of the different expressions of the nine prognostic related lncRNAs among tumor and normal sample. The ordinate represents the lncRNAs, while red represents high expression and blue represents low expression. The abscissa represents the sample. *p < 0.05, **p < 0.01, ***p < 0.001.

By combining the m^6^A-related lncRNA expression data with the survival data, then using univariate Cox regression, we obtained nine prognostic m^6^A-related lncRNAs: LINC02657, NSMCE1−DT, AC139149.1, ZKSCAN2−DT, AC156455.1, ZEB1−AS1, AP001619.1, AL391422.4, and ATP2B1−AS1 (*p* < 0.001; if *p* < 0.05, there were too many lncRNAs). All of them were high-risk lncRNAs (Supplementary Table S1). The expression of AC156455.1 in tumor tissue was significantly higher than that of the normal group. The results were visualized by forest plots, box plots, and heat maps (Figures 2A–C).

### The Independent Prognostic Model of Seven m^6^A-Related LncRNAs

**FIGURE 3:**
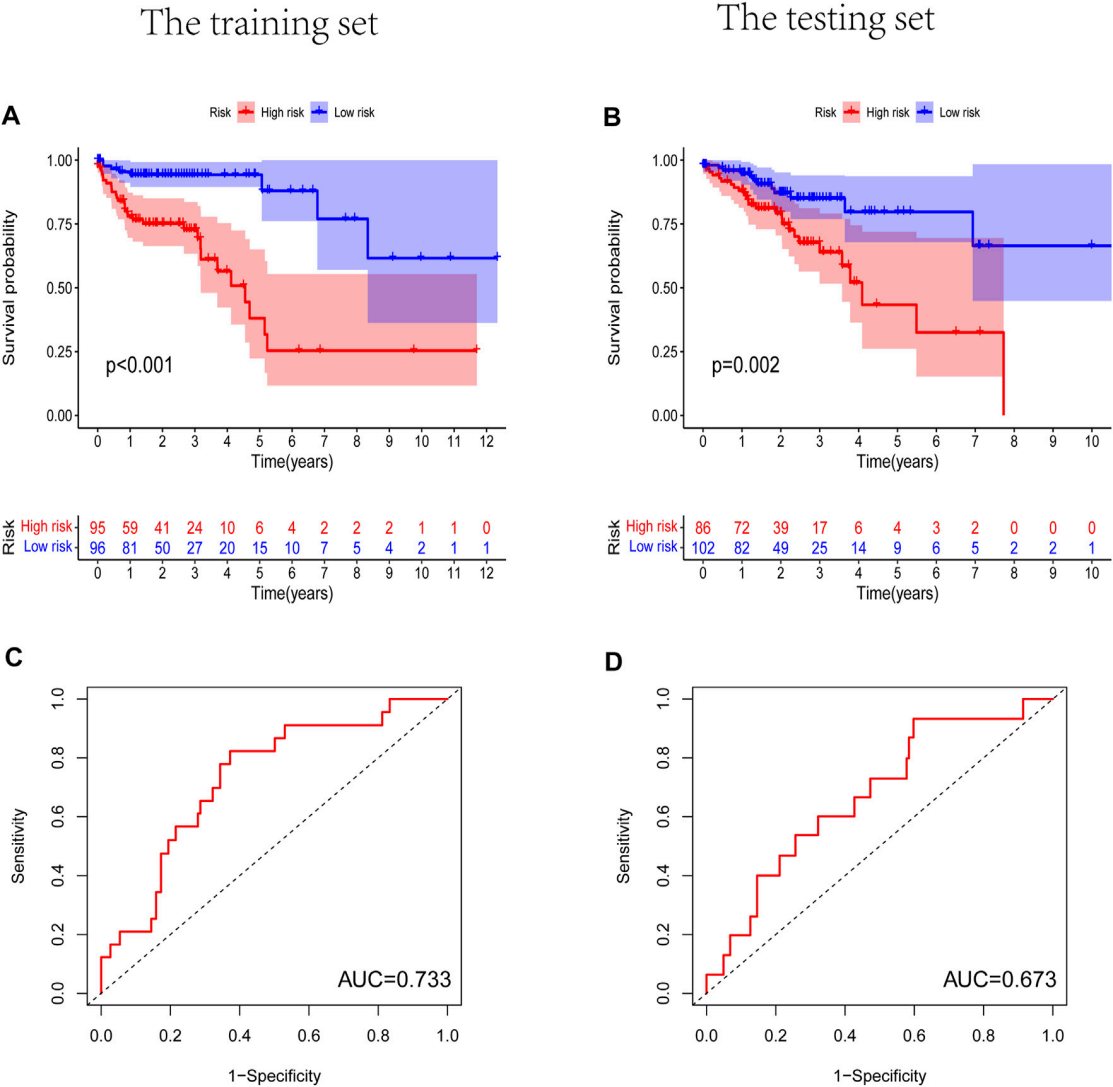
The Kaplan-Meier survival curve analysis and the area under the ROC curve (AUC). **(A,B)** Survival curve of high- and low- riskgroups. (*p* < 0.01). **(C,D)** ROC curve to evaluate the accuracy of our model. (AUC > 0.5). **(A,C)** Training dataset, **(B,D)** testing dataset.

**FIGURE 4:**
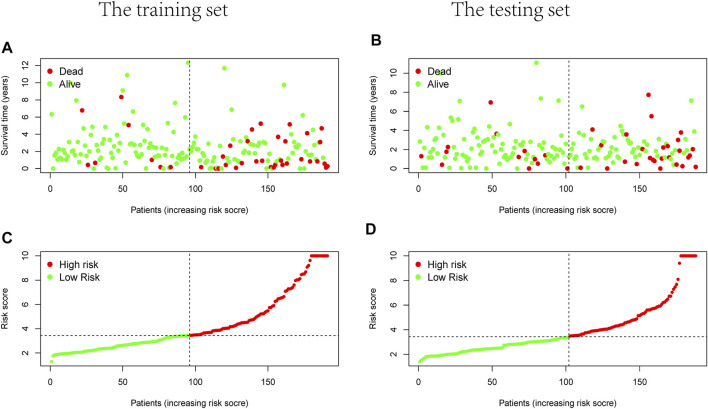
Survival and risks state map. **(A,B)** Survival state of each patient, **(C,D)** risk state of each patient. **(A,C)** Training dataset, **(B,D)** testing dataset.

**FIGURE 5:**
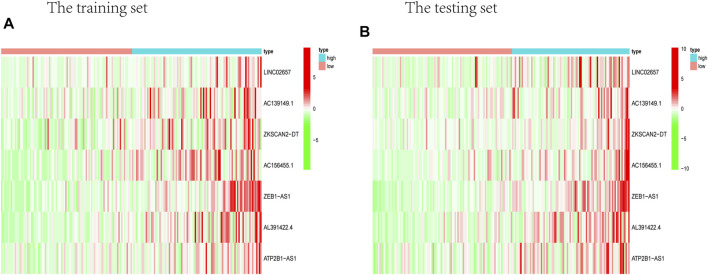
Heat map about the expression of m6A-related prognostic lncRNAs. **(A)** Training group. **(B)** Testing group. ZEB1-AS1 was significantly higher in both testing and training high-risk groups than low-risk ones.

**FIGURE 6:**
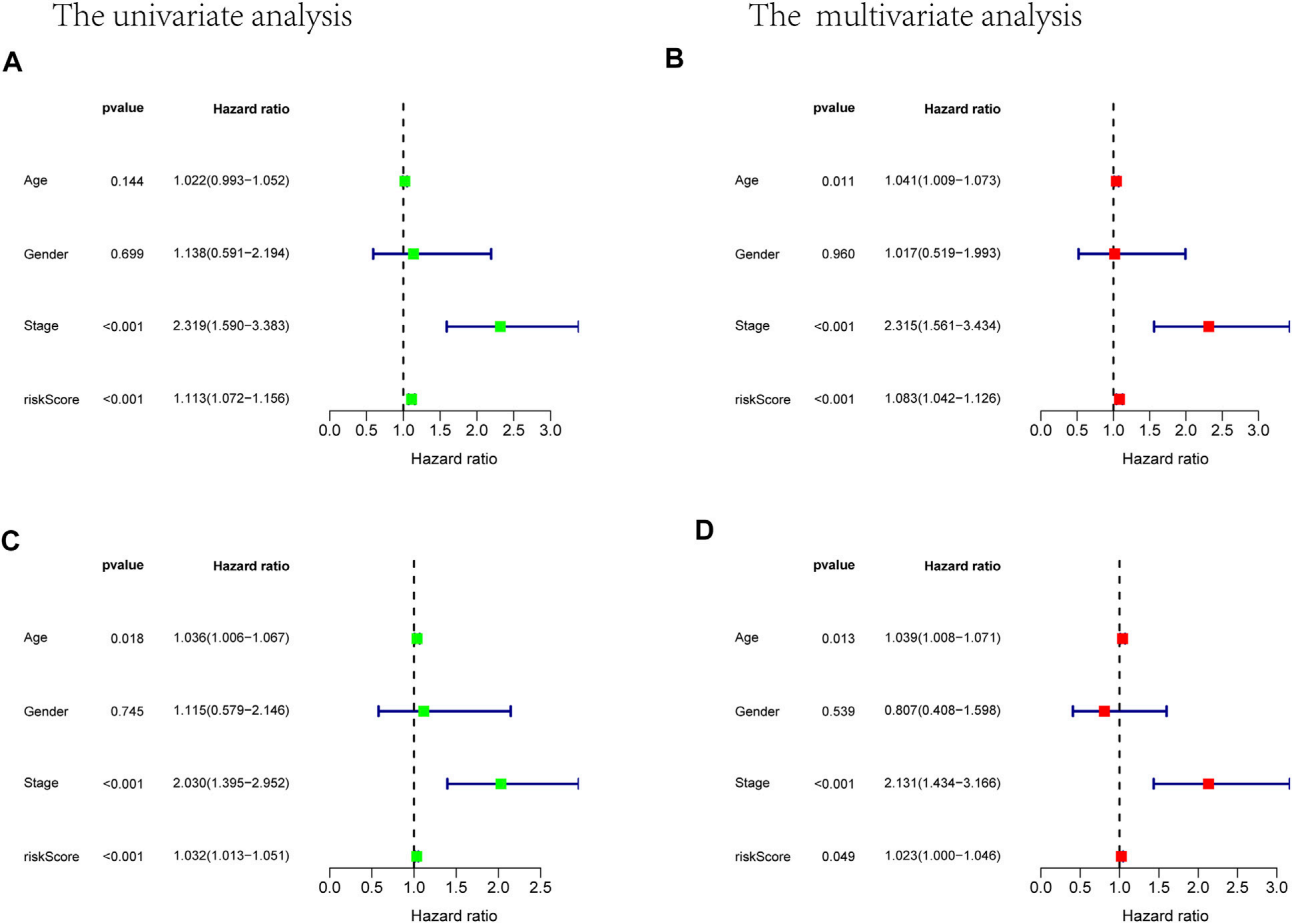
Univariate and multivariate Cox analysis considering riskscore, age, gender, and stage, in training cohort **(A,B)** and test cohort **(C,D)**.

Prognostic m^6^A-related lncRNA samples were divided into training and test datasets (50% in each group). Through the R packages and Lasso regression, using the cross-validation method to optimize the model, we finally obtained the independent prognostic model consisting of seven lncRNAs, model formula: risk score = LINC02657 * 0.246632699492067 + AC139149.1 * 0.276937064691493 + ZKSCAN2-DT * 0.016797173163626 + AC156455.1 * 0.187570142850178 + ZEB1-AS1 * 0.569626111717306 + AL391422.4 * 0.537921082314907 + ATP2B1-AS1 * 0.555444251652824 **(** Supplementary Figures S1A, B). There were survival differences between the high- and low-risk groups in the training and test groups, with the survival curve drawn by the R package (*p* < 0.05). The high-risk group had a low survival rate (Figures 3A, B). In addition, the area under the curve (AUC) was 0.733 and 0.673 in the training dataset and testing dataset for overall survival (OS) at 1 year (Figures 3C, D), revealing the high accuracy of this model in predicting the prognosis. Through the risk curve of heat map, survival state, and risk state map, we found that more deaths occurred in the high-risk group (Figures 4A–D). The expression of the all prognostic lncRNAs was higher in the high-risk group among both training and testing datasets. The expression of the lncRNA ZEB1−AS1 was significantly higher in the high-risk groups (Figures 5A, B). By univariate and multivariate Cox regression, the risk score was identified as an independent prognostic indicator (Figures 6A–D), verifying the independence of the model.

### Relationship Between Different Clusters or High-Low Risk Groups With Clinicopathological Features

Prognostic m^6^A-related lncRNAs were divided into two types through consistent clustering analysis **(** Supplementary Figures S2A–D). Differences in survival analysis were found between the two groups (*p* < 0.01).

**FIGURE 7:**
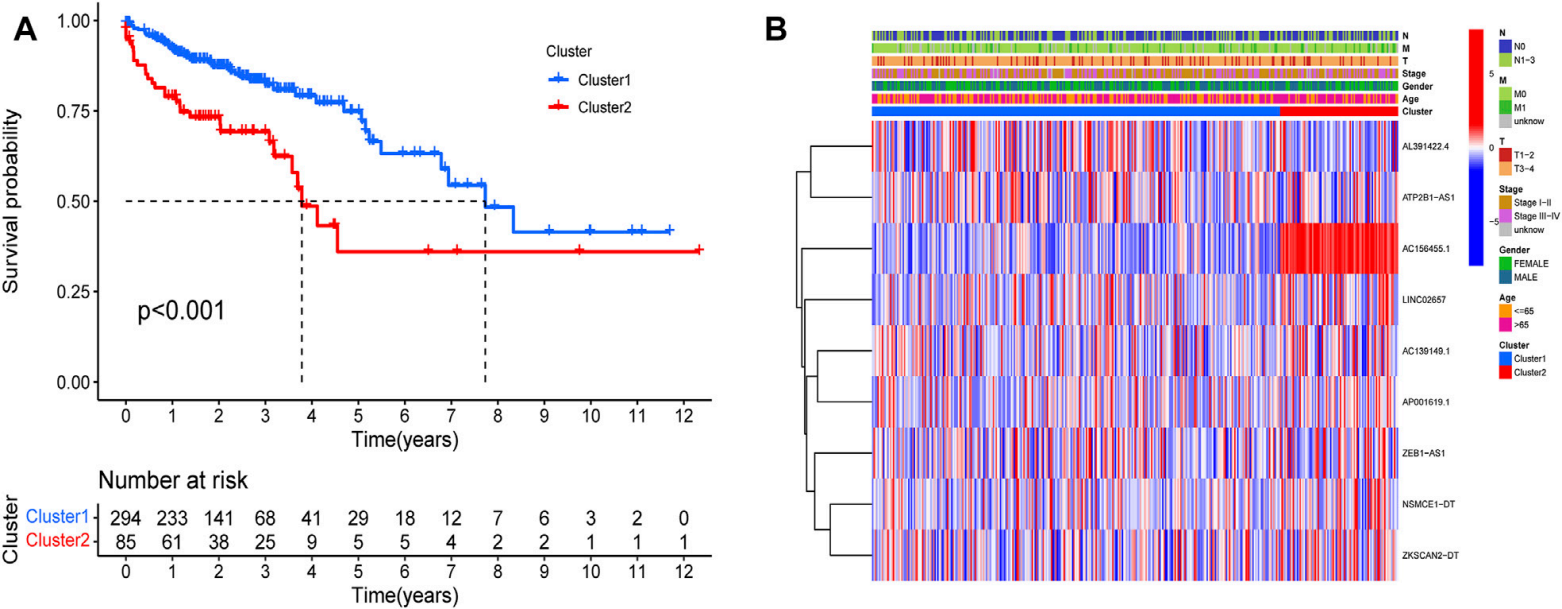
Kaplan-Meier curves of OS for two clusters in COAD. Relationships between lncRNAs of clusters and clinical features. **(A)** Survival analysis of the lncRNAs in two clusters. The survival rate of type 2 was obviously low (*p* < 0.001). **(B)** Heatmap of the two clusters along with clinicopathological characteristics. AC156455.1 expression was significantly higher in type 2.

The survival rate of type 2 was low, suggesting a poor prognosis (Figure 7A). We analyzed the correlation between the two subtypes with clinical characteristics, visualized by Figure 7B. AC156455.1 expression was found to be significantly higher in type 2, suggesting that AC156455.1 may be predominantly related to the occurrence and development of COAD.

**FIGURE 8:**
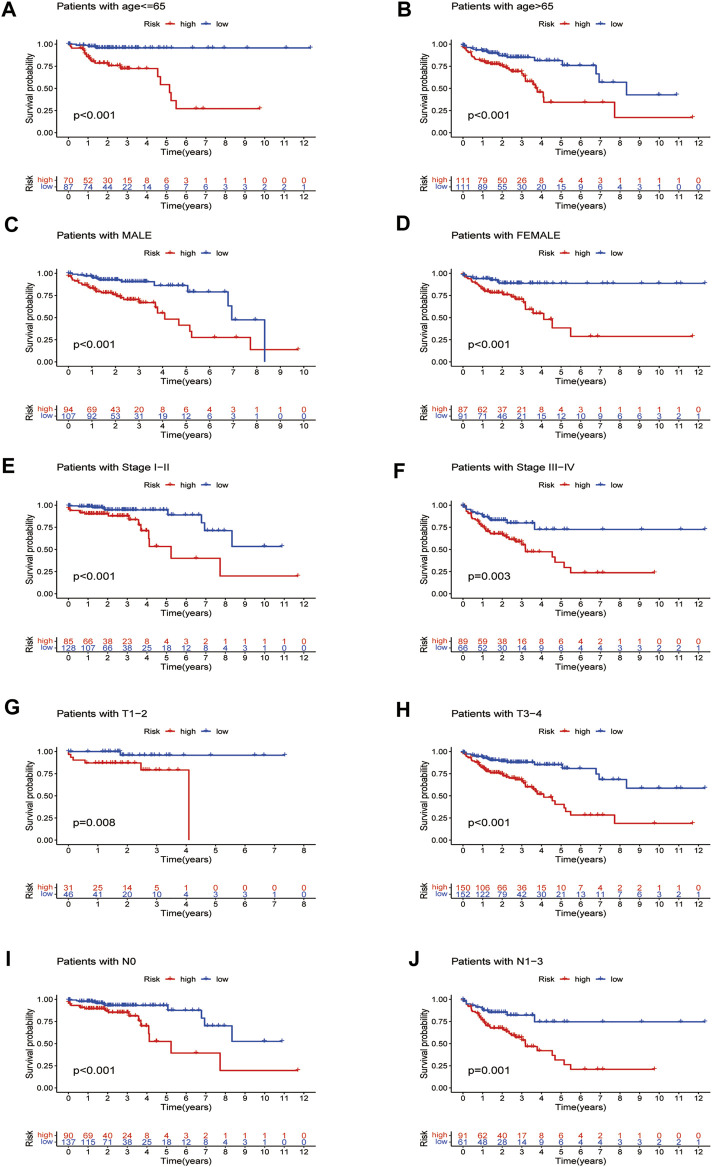
Survival curves for model validation (our prognostic model applied to different clinical groups: age, gender, stage, and T and N staging, *p* < 0.05).

**FIGURE 9:**
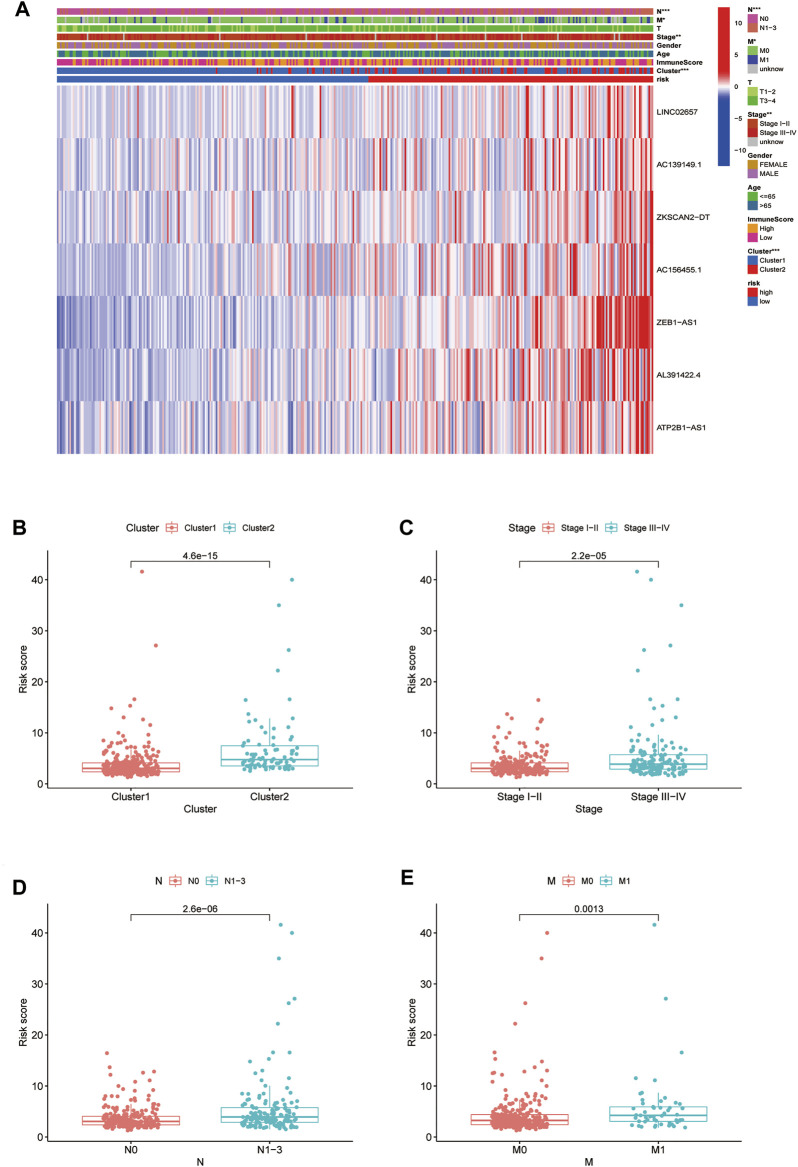
Relationship between high-low risk groups and clinicopathological features. **(A)** Heatmap of correlation analysis between riskscore and clinical traits; **(B–E)** boxplot of relationship analysis of riskscore and clinical features (clusters, clinical traits such as stage,and N and M staging were closely related to riskscore, *p* < 0.05.

By verifying the prognostic model on the clinical traits, it was found that the model applied to clinical characteristics such as age, gender, stage, and T and N staging (Figures 8A–J). Analyzing differences of different clusters, clinicopathological characteristics, immune scores, and the lncRNA expression of the high- and low-risk groups, we discovered differences in clusters and clinical traits (stages, N and M staging) in the high- and low-risk groups (Figures 9A–E). The proportion of cluster two in the high-risk group was high, while the low survival rate indicated that the high-risk group was accompanied by a poor prognosis. Advanced clinical features such as stage III–IV, N1-3, and M1 occupied more in the high-risk groups, showing the higher risk attached to the worse clinical stage. The results of the heat map indicated that all prognostic lncRNAs were high-risk markers. The expression of ZEB1-AS1 was significantly increased in the high-risk group both on the risk curve and on the heat map. Hence, further studies should be conducted to explore the prognostic value of lncRNA ZEB1-AS1 in COAD.

### Relationship Between Different Clusters or High-Low Risk Groups With Immune Cell Infiltration

**FIGURE 10:**
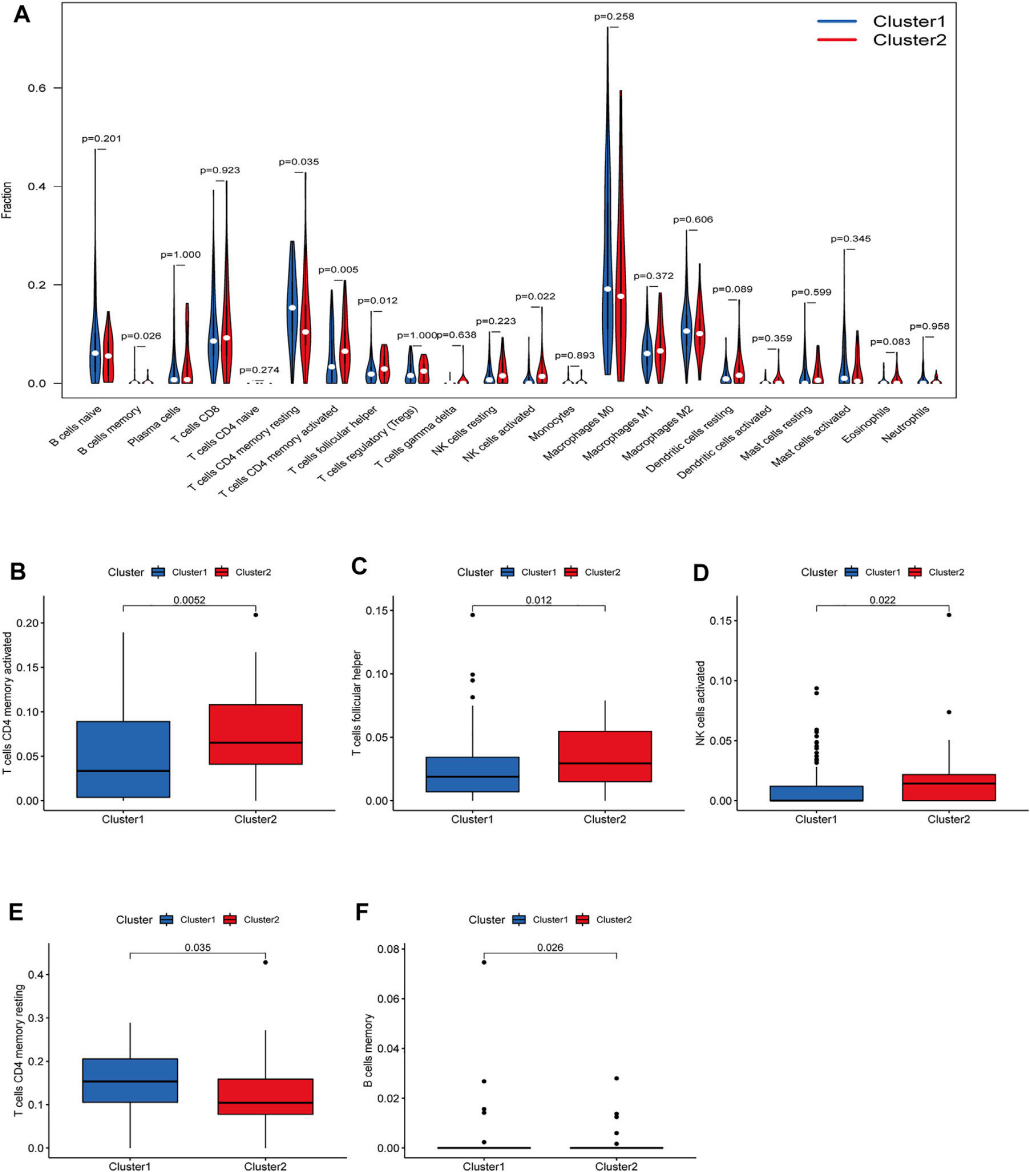
Diferent analysis of immune cell infltration in the two clusters. **(A)** Vioplot; **(B–F)** Boxplot. CD4 memory activated T cells, follicular helper T cells and a1186ctivated NK cells were upregulated in type 2, which had a low survival rate.

**FIGURE 11:**
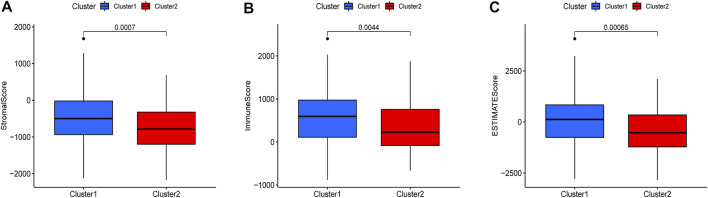
Different expressions of ESTIMATEscore, Immunescore, and Stromalscore in two clusters (*p* < 0.05).

**FIGURE 12:**
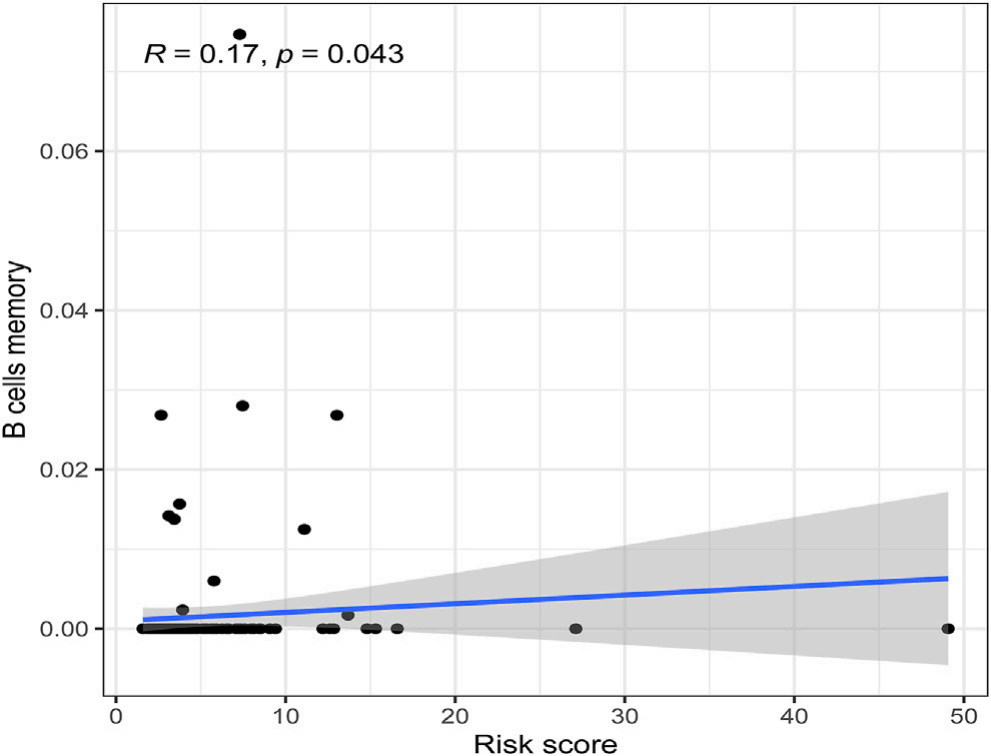
Scatterplots of correlation analysis of riskscore and immunecells. Memory B cells were correlated with the riskscore (*p* < 0.05, R > 0), indicating that its content was positively correlated with the risk of the patients.

There were five types of immune cells expressed differently in two different clusters: CD4 memory activated T cells, follicular helper T cells, activated natural killer (NK) cells, CD4 memory T cells, and memory B cells (Supplementary Table S2). The first three were upregulated in type 2, which had a low survival rate. They may be relevant to poor prognosis and provide some reference for immunotherapy. T cells CD4 memory resting was upregulated in type 1 (Figures 10A–F). There were differences in the immune score, stromal score, and estimate score between type 1 and type 2; all had low scores in type 2. The lower the score was, the higher the tumor purity was, which may correlate with poor prognosis (Figures 11A–C). We got risk scores of the samples according to the prognostic model formula. We explored which immune cells were associated with patients' risk using limma, ggplot2 ggpubr, ggextra packages, and Spearman test. Finally, memory B cells were correlated with the risk score (*p* < 0.05, R > 0), indicating that its content was positively correlated with the risk of the patients. The higher the content of memory B cells, the higher risk the patient had (*R* = 0.17, *p* = 0.043), visualized by a scatter diagram (Figure 12).

### Validation of GEO Dataset

**FIGURE 13:**
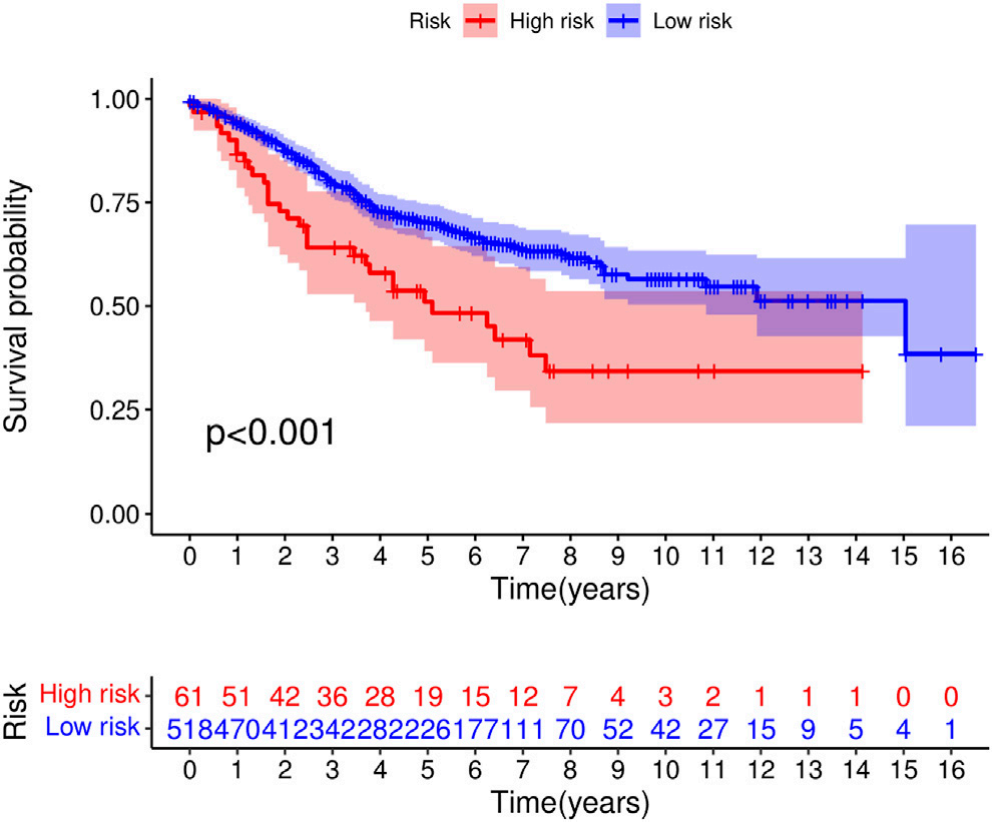
Survival curve of high- and low-risk groups from GEO dataset (*p* < 0.001).

We obtained the GSE39582 data set from GEO database which contained 579 samples and then obtained the expression levels of seven m^6^A-related LncRNAs as well as the survival data of the samples. According to the model, we calculated the risk score and obtained the high- and low-risk groups based on the median risk scores of the training dataset of TCGA data. Finally, we found there were survival differences between the high- and low-risk groups, *p* < 0.001 (Figure 13). Our model was validated in the GEO dataset.

### Validation of the Expression Level of Screened m^6^A-Related LncRNAs by qRT-PCR

**FIGURE 14:**
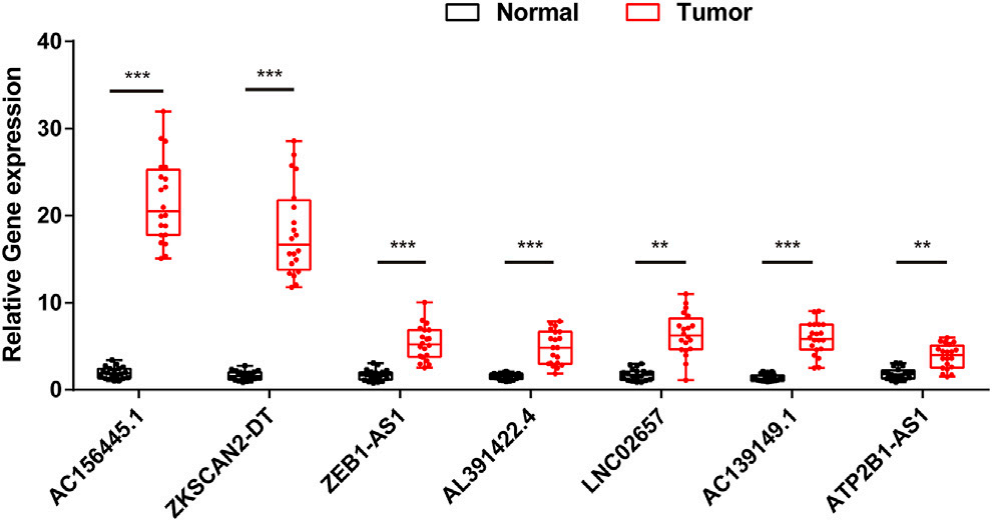
The expression levels of seven m6A-related lncRNAs in 20 paired COAD and matched adjacent normal tissues were examined by qRT-PCR. *:*p* < 0.05, **:*p* < 0.01, ***:*p* < 0.001.

In order to further validate the feasibility of the prognostic signature, we performed qRT-PCR experiment to identify the expression levels of the seven lncRNAs in clinical tissue samples. Finally, we found the expression of these seven lncRNAs in tumor tissues was significantly higher than that in tumor-adjacent normal tissues (Figure 14), especially ZKSCAN2-DT and AC156455.1, consistent with the TCGA dataset. Collectively, this further validated the stability and reliability of the m6A-related lncRNAs prognostic signature. *β*-Actin was chosen as the internal control. The primers used are listed in Table 1.

**TABLE 1:** Primers used in qRT-PCR.

Primer	Sequence	Primer length	Tm	Product size
AL391422.4-F-H	GTG​TGA​GTG​TGG​TAT​GGC​TGT​GTC	24	60.4	134 bp
AL391422.4-R-H	TGG​AAG​GCG​GAG​GTT​GTA​GTG​AG	23	61.5	
ATP2B1-AS1-F-H	ACG​CCC​CTC​CCT​TTC​TTC​CTT​C	22	62.3	146 bp
H ATP2B1-AS1-R-H	CCT​CCT​GCA​CCA​ACA​CGT​CAT​G	22	61.5	
AC156455.1-F-H	TCA​TCT​GAC​CTC​CTG​GCA​ACC​C	22	62.1	135 bp
AC156455.1-R-H	TCC​GAA​GCC​TCC​TTC​ACT​GAG​TC	23	61.2	
ZKSCAN2-DT-F-H	TCA​TCT​GAC​CTC​CTG​GCA​ACC​C	25	59.4	132 bp
ZKSCAN2-DT-R-H	TCC​GAA​GCC​TCC​TTC​ACT​GAG​TC	23	60.9	
AC139149.1-F-H	TGT​AAT​CAG​TAG​AGC​AGG​GCA​GAG​G	25	60.4	88 bp
AC139149.1-R-H	AGA​GAC​AGA​GAA​CCA​GGA​CGG​AAG	24	60.4	
ZEB1-AS1-F-H	TGG​CAG​GAC​TCA​GAG​CTA​AGG​TAT​C	25	60.2	105 bp
ZEB1-AS1-R-H	ACA​TCT​GTC​AGC​CGA​TGC​TTC​TTG	24	59.7	
LNC02657-F-H	GCA​AGA​GAG​AAG​ACA​GTG​GGT​GAA​G	25	59.8	135 bp
LNC02657-R-H	ATT​TGT​GCC​GTG​ACT​CTG​GGA​AC	23	60.2	
β-actin-F	GGC​TGT​ATT​CCC​CTC​CAT​CG	20	61.8	154 bpzz
β-actin-R	CCA​GTT​GGT​AAC​AAT​GCC​ATG​T	22	61.1	

## Discussion

Aberrant m^6^A modifications are closely related to the onset and progression of various cancers, including CRC (Liu et al., 2018; Gong et al., 2019; Wang et al., 2020; Wang et al., 2021; Zhang and Zhang., 2021). Nowadays, lncRNAs are reported to act essential roles in the development of tumors, especially in epigenetic regulation, protein interaction, and RNA metabolism. Emerging researches on N^6^-methyladenosine of lncRNA are performed to provide prognosis-related biomarkers and novel therapeutic targets for various carcinomas (Li et al., 2021a; Chen et al., 2021; Han et al., 2021). In our study, we obtained nine prognostic m^6^A-related lncRNA of COAD. All of them were high risk, meaning that the genes with higher expression have a worse prognosis. We finally got the independent prognostic model consisting of seven m^6^A lncRNAs. There was a difference in survival probability between high- and low-risk group samples, in both training and testing groups. The high-risk group has a low survival rate. Moreover, the AUC showed that the model accurately predicts the prognosis. Through the risk curve, we found that more deaths occurred in the high-risk group.

Furthermore, we explored the relationship between risk score and clinical characteristics and found that advanced clinical features such as stage III–IV, N1-3, and M1 occupied more in the high-risk groups. It showed that the high-risk score was highly correlated with malignant clinical characteristics. Therefore, the model can be used as a new potential prognostic biomarker of COAD.

In the study, we found two special lncRNAs, AC156455.1 and ZEB1−AS1. Yang et al. constructed a risk signature of seven lncRNAs (LINC00460, AL139351.1, AC156455.1, AL035446.1, LINC02471, AC022509.2, and LINC01606) for clear cell renal cell carcinoma patients (Yang et al., 2021). AC156455.1 was included in Yang's signature and also existed in our model. The level of AC156455.1 expression in tumor tissues was significantly higher than that of the normal group. Meanwhile, the expression level of AC156455.1 was significantly higher in type 2, which had a low survival rate. Being of high risk, AC156455.1 was associated with the malignancy of COAD, suggesting that it could be further studied for specific prognostic value. The other identified lncRNA was ZEB1−AS1, the expression of which was significantly higher in high-risk groups of both test and training samples and could also be further studied for the prognosis of COAD.

Zheng et al. identified a risk model of four m^6^A-related lncRNA predicting the OS and therapeutic value of ovarian cancer (OC). LncRNA CACNA1G-AS1 was identified to be upregulated in 30 OC specimens and 3 OC cell lines, while its knockdown restrained the multiplication capacity of OC cells (Zheng et al., 2021). Zheng and collages verified this lncRNA in OC samples, and tumor cell lines revealed its potential prognostic and therapeutic value. In the same way, we could conduct further research on ZEB1−AS1 and AC156455.1, which we found to be closely correlated with COAD.

Yang et al. found that through the m^6^A modification of METTL3-mediated MYC mRNA, HBXIP can lead to tumorigenesis of GC (Yang Z. et al., 2020). Different enzymes that participate in m^6^A modifications can lead to progression in different cancers (Yang et al., 2020a), while the underlying mechanisms, such as the involved signaling pathways, need further research.

In addition, methylation is a reversible process, and demethylation can be used in tumor therapy. ALKBH5, one of the demethylases, could affect tumor progression by regulating lncRNA demethylation. In a research about pancreatic cancer, the expression of ALKBH5 was found downregulated in tumor cells and could inhibit cells by demethylating the lncRNA KCNK15-AS1, which can be used in prognostics and therapy strategies (He et al., 2018).

When it comes to the point above, by what methylation mechanism does our prognostic lncRNAs act in, whether it can be mediated by MettL3, and what the signaling pathway is, whether it can be demethylated, all these need further research.

Yu and Zhu found immune cell infiltration related to m6A-correlated lncRNA in GC can provide novel therapeutic value. The high-risk scores indicated a lower purity of tumor cells, as well as a higher density of immune-related cells (Yu and Zhu, 2021). Gaudreau et al. investigated the correlation between neoadjuvant chemotherapy (NCT) and the immune microenvironment (IME) in resectable NSCLC (non-small cell lung cancer) and discovered NCT was closely related to the cell infiltration increase of cytotoxic CD8^+^ T cells and CD20 ^+^ B cells (Gaudreau et al., 2021). In our study, we found five kinds of immune cells expressed differently in different types of lncRNA, that is, CD4 memory activated T cells, follicular helper T cells, activated NK cells, CD4 memory T cells, and memory B cells. The first three were upregulated in type 2, which had a low survival rate. They may be relevant to poor prognosis. There were differences in the immune score, stromal score, and estimate score between type 1 and type 2, all of which had low scores in type 2. The lower the score was, the higher the tumor purity was. This may be correlated with a poor prognosis. Memory B cells were correlated with the risk score. We found that m^6^A-related lncRNAs were associated with immune cell infiltration in COAD, which could assist us in exploring new therapeutic targets. Although tumor immunotherapy has made rapid progress in recent years, the overall therapeutic effect of COAD is not satisfactory. So it is necessary to develop multimode therapy and biointegration targets. Further investigation is needed between m^6^A-related lncRNAs and COAD.

In summary, in this study, we obtained seven m^6^A lncRNAs signature and immune infiltration results. Besides, we have verified the prognostic model by GEO dataset, and the expression level of lncRNAs was further verified by in vitro experiments. This suggests the signature could be used as a new potential and promising biomarker and provide an individual treatment strategy for COAD. Nevertheless, the m^6^A methylation mechanism of these lncRNAs is still unknown. Furthermore, the specific correlation between m^6^A-related lncRNA and immune cells should be thoroughly explored.

## Data Availability

Publicly available datasets were analyzed in this study. These data can be found here: The Cancer Genome Atlas (TCGA) (https://portal.gdc.cancer.gov/).
