# Supplementary figures and images for: Potential Prognostic Value of a Seven m6A-Related LncRNAs Signature and the Correlative Immune Infiltration in Colon Adenocarcinoma
Source: Front Genet. 2021 Dec 22;12:774010. doi: 10.3389/fgene.2021.774010 (PMC8727540; doi:10.3389/fgene.2021.774010)

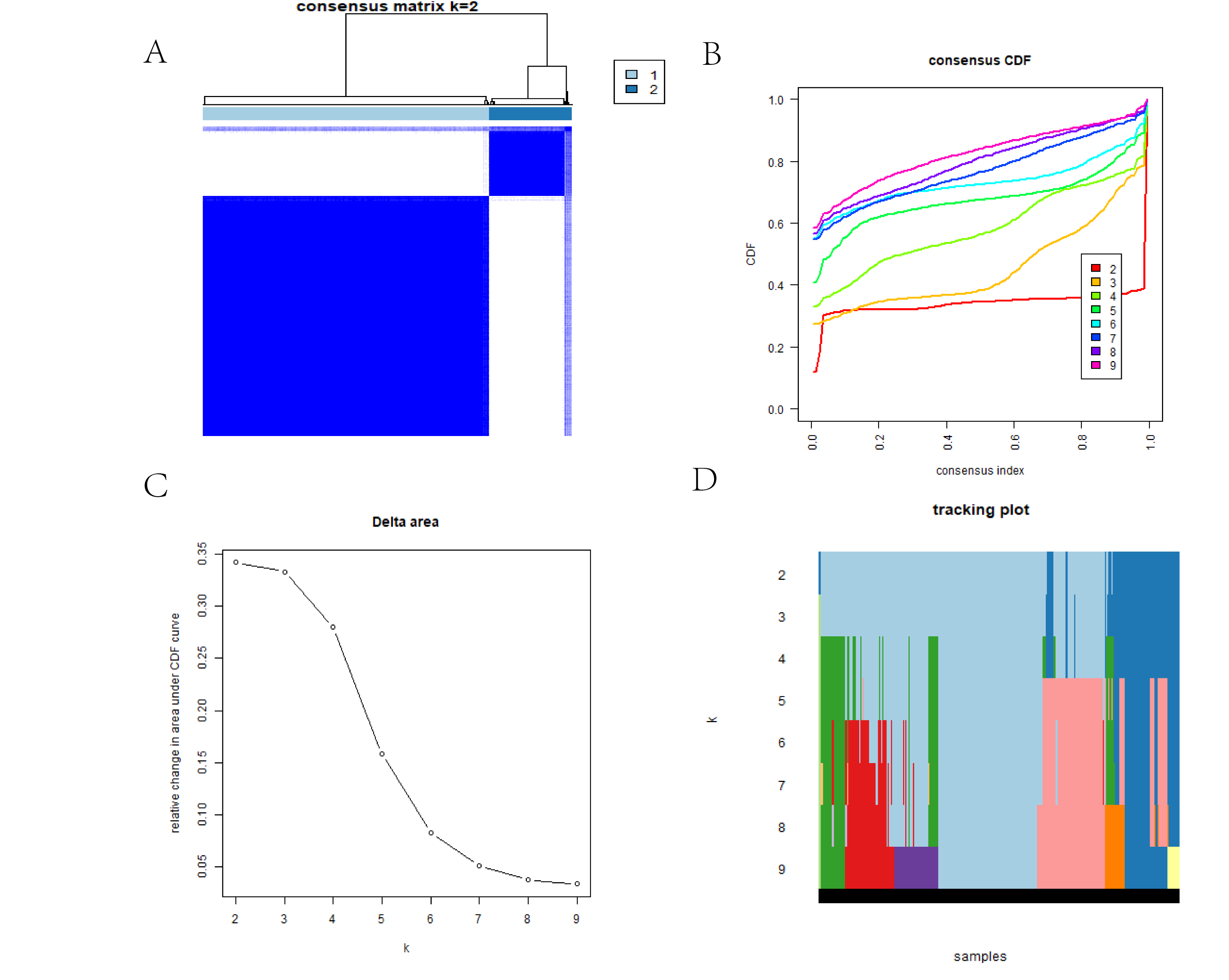

Supplement: Supplementary file 1 [file Image2.TIF]

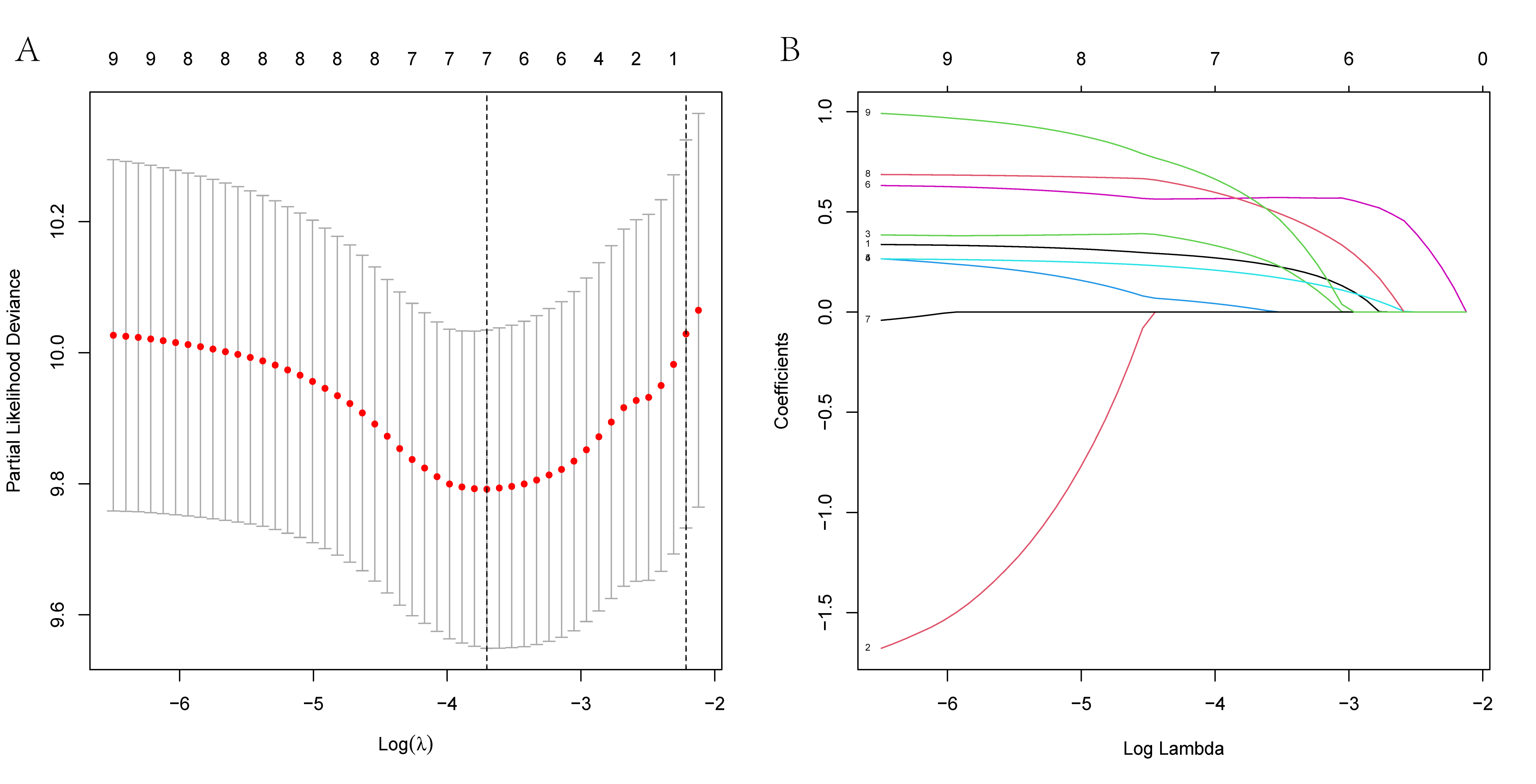

Supplement: Supplementary file 2 [file Image1.TIF]
