# Supplementary material for: Potential Prognostic Value of a Seven m6A-Related LncRNAs Signature and the Correlative Immune Infiltration in Colon Adenocarcinoma
Source: Front Genet. 2021 Dec 22;12:774010. doi: 10.3389/fgene.2021.774010 (PMC8727540; doi:10.3389/fgene.2021.774010)
Supplement: Supplementary file 3 [file DataSheet2.docx]

| **gene** | **HR** | **HR.95L1q** | **HR.95H** | **P-value** |
| --- | --- | --- | --- | --- |
| LINC02657 | 2.04532 | 1.42463 | 2.93642 | 0.00010 |
| NSMCE1-DT | 15.24102 | 3.14004 | 73.97618 | 0.00072 |
| AC139149.1 | 3.983387 | 1.93249 | 8.21083 | 0.00018 |
| ZKSCAN2-DT | 1.38997 | 1.19851 | 1.61202 | 1.33231e-05 |
| AC156455.1 | 1.18854 | 1.07639 | 1.31237 | 0.00063 |
| ZEB1-AS1 | 2.35320 | 1.63457 | 3.38778 | 4.16591e-06 |
| AP001619.1 | 2.42059 | 1.51341 | 3.87157 | 0.00022 |
| AL391422.4 | 2.33576 | 1.46654 | 3.72016 | 0.00035 |
| ATP2B1-AS1 | 10.44076 | 2.66485 | 40.90644 | 0.00076 |

Supplementary TABLE 1 The nine m6A-related prognostic lncRNAs

Supplementary TABLE 2 The five kinds of immune cells expressed differently in the two different clusters

| **Cell** | **P-value** |
| --- | --- |
| B cells memory | 0.02591 |
| T cells CD4 memory resting | 0.03453 |
| T cells CD4 memory activated | 0.00516 |
| T cells follicular helper | 0.01176 |
| NK cells activated | 0.02160 |

TABLE 1 Primers used in qRT-PCR.

| **Primer** | **Sequence** | **Primer length** | **Tm** | **Product size** |
| --- | --- | --- | --- | --- |
| AL391422.4-F-H | GTGTGAGTGTGGTATGGCTGTGTC | 24 | 60.4 | 134bp |
| AL391422.4-R-H | TGGAAGGCGGAGGTTGTAGTGAG | 23 | 61.5 |  |
| ATP2B1-AS1-F-H | ACGCCCCTCCCTTTCTTCCTTC | 22 | 62.3 | 146bp |
| H ATP2B1-AS1-R-H | CCTCCTGCACCAACACGTCATG | 22 | 61.5 |  |
| AC156455.1-F-H | TCATCTGACCTCCTGGCAACCC | 22 | 62.1 | 135bp |
| AC156455.1-R-H | TCCGAAGCCTCCTTCACTGAGTC | 23 | 61.2 |  |
| ZKSCAN2-DT-F-H | TCATCTGACCTCCTGGCAACCC | 25 | 59.4 | 132bp |
| ZKSCAN2-DT-R-H | TCCGAAGCCTCCTTCACTGAGTC | 23 | 60.9 |  |
| AC139149.1-F-H | TGTAATCAGTAGAGCAGGGCAGAGG | 25 | 60.4 | 88bp |
| AC139149.1-R-H | AGAGACAGAGAACCAGGACGGAAG | 24 | 60.4 |  |
| ZEB1-AS1-F-H | TGGCAGGACTCAGAGCTAAGGTATC | 25 | 60.2 | 105bp |
| ZEB1-AS1-R-H | ACATCTGTCAGCCGATGCTTCTTG | 24 | 59.7 |  |
| LNC02657-F-H | GCAAGAGAGAAGACAGTGGGTGAAG | 25 | 59.8 | 135bp |
| LNC02657-R-H | ATTTGTGCCGTGACTCTGGGAAC | 23 | 60.2 |  |
| β-actin-F | GGCTGTATTCCCCTCCATCG | 20 | 61.8 | 154bpzz |
| β-actin-R | CCAGTTGGTAACAATGCCATGT | 22 | 61.1 |  |
