# Supplementary material for: Potential Prognostic Value of a Seven m6A-Related LncRNAs Signature and the Correlative Immune Infiltration in Colon Adenocarcinoma
Source: Front Genet. 2021 Dec 22;12:774010. doi: 10.3389/fgene.2021.774010 (PMC8727540; doi:10.3389/fgene.2021.774010)
Supplement: Supplementary file 4 [file DataSheet1.docx]

FIGURE 1 Study flow chart of the integration analysis.

FIGURE 2. Nine prognostic m^6^A-related lncRNAs of COAD. (A) Forest map of nine prognostic m^6^A-related lncRNAs by univariate cox regression analysis. Red represents high risk, while green represents low risk, all of them were high-risk. (B) Boxplot of the diferent expression of the nine prognostic related lncRNAs among tumor and normal tissue; the expression of AC156455.1 in tumor tissue was significantly higher than that of the normal group. (C) Heatmap of the diferent expression of the nine prognostic related lncRNAs among tumor and normal sample. The ordinate represents the lncRNAs, while red represents high expression and blue represents low expression. The abscissa represents the sample. *:P<0.05, **:P<0.01, ***:P<0.001.

FIGURE 3. LASSO regression analysis of prognostic m6A-related lncRNAs. (A) Lasso coefficient values of nine m^6^A-related lncRNAs in COAD. The vertical dashed lines are at the optimal log (lambda) value. (B) Profiles of Lasso coefficients.

FIGURE 4 The KM survival curve analysis and the area under the ROC curve (AUC). ( A+B): Survival curve of high- and low-risk groups.(P<0.01). (C+D): ROC curve to evaluate the accuracy of our model. (AUC>0.5). A+C represent training dataset, B+D represent testing dataset.

FIGURE 5 Survival state and Risk state map. (A+B) Survival state of each patient, (C+D) Risk state of each patient, (A+C) represent training dataset, (B+D) represent testing dataset.

FIGURE 6. Heatmap about the expression of m^6^A-related prognostic lncRNAs. (A) represents training group. (B) represents testing group. ZEB1−AS1 was significantly higher in both testing and training high-risk groups than low-risk ones.

FIGURE 7 Univariate and multivariate Cox analysis considering riskscore , age, gender and stage, in training cohort (A+B) and test cohort (C+D).

FIGURE 8 Prognostic m^6^A-related lncRNAs were divided into two clusters through consistent clustering analysis, when K=2, there were least cross-mixing part between the two types and the CDF value was lowest.

FIGURE 9 Kaplan–Meier curves of OS for two clusters in COAD. Relationships between lncRNAs of clusters and clinical features. (A) Survival analysis of the lncRNAs in two clusters. The survival rate of type 2 was obviously low (P<0.001). (B) Heatmap of the two clusters along with clinicopathological characteristics. AC156455.1 expression was significantly higher in type 2.

FIGURE 10 Survival curves for model validation. (our prognostic model applied to different clinical groups: age, gender, stage, T and N staging, P<0.05.)

FIGURE 11 Relationship between high-low risk groups with clinicopathological features. (A) Heatmap of correlation analysis between risk score and clinical traits; (B-E) Boxplot of relationship analysis of risk score and clinical features (clusters, clinical traits such as stage, N and M staging were closely related to risk score, P<0.05).

FIGURE 12 Diferent analysis of immune cell infltration in the two clusters. (A) Vioplot; (B) Boxplot. CD4 memory activated T cells, follicular helper T cells and activated NK cells were upregulated in type 2, which had a low survival rate.

FIGURE 13 Different expression of ESTIMATEscore, Immunescore and Stromalscore in two clusters (P < 0.05).

FIGURE 14 Scatter plots of correlation analysis of risk score and immune cells. Memory B cells were correlated with the risk score (P < 0.05, R > 0), indicating that its content was positively correlated with the risk of the patients.

Supplementary Figure 1. LASSO regression analysis of prognostic m6A-related lncRNAs. (A) Lasso coefficient values of nine m^6^A-related lncRNAs in COAD. The vertical dashed lines are at the optimal log (lambda) value. (B) Profiles of Lasso coefficients.

Supplementary Figure 2 Prognostic m^6^A-related lncRNAs were divided into two clusters through consistent clustering analysis, when K=2, there were least cross-mixing part between the two types and the CDF value was lowest.
